# Organized cannabinoid receptor distribution in neurons revealed by super-resolution fluorescence imaging

**DOI:** 10.1038/s41467-020-19510-5

**Published:** 2020-11-11

**Authors:** Hui Li, Jie Yang, Cuiping Tian, Min Diao, Quan Wang, Simeng Zhao, Shanshan Li, Fangzhi Tan, Tian Hua, Ya Qin, Chao-Po Lin, Dylan Deska-Gauthier, Garth J. Thompson, Ying Zhang, Wenqing Shui, Zhi-Jie Liu, Tong Wang, Guisheng Zhong

**Affiliations:** 1 iHuman Institute, ShanghaiTech University, 201210 Shanghai, China; 2 University of the Chinese Academy of Sciences, 100049 Beijing, China; 3 School of Life Science and Technology, ShanghaiTech University, 201210 Shanghai, China; 4 Department of Medical Neuroscience, Dalhousie University, Halifax, NS B3H 4R2 Canada

**Keywords:** Super-resolution microscopy, Cellular neuroscience

## Abstract

G-protein-coupled receptors (GPCRs) play important roles in cellular functions. However, their intracellular organization is largely unknown. Through investigation of the cannabinoid receptor 1 (CB_1_), we discovered periodically repeating clusters of CB_1_ hotspots within the axons of neurons. We observed these CB_1_ hotspots interact with the membrane-associated periodic skeleton (MPS) forming a complex crucial in the regulation of CB_1_ signaling. Furthermore, we found that CB_1_ hotspot periodicity increased upon CB_1_ agonist application, and these activated CB_1_ displayed less dynamic movement compared to non-activated CB_1_. Our results suggest that CB_1_ forms periodic hotspots organized by the MPS as a mechanism to increase signaling efficacy upon activation.

## Abstract (web summary)

Despite the importance of G-protein-coupled receptors in many cellular functions, their intracellular organisation is largely unknown. The authors identified periodically repeating clusters of cannabinoid receptor 1 hotspots within neuronal axons that are dynamically regulated by CB_1_ agonists.

## Introduction

G-protein-coupled receptors (GPCRs) are a large family of membrane proteins that play important roles in cellular functions by initializing a variety of intracellular processes via neurotransmitters and hormone signaling. To fulfill their function, GPCRs are likely anchored by cellular skeletal structures facilitating their interactions with intracellular protein complexes. Therefore, their membrane organization and relationship with skeleton proteins, which are still largely unknown, is likely critical for their cellular function. In addition, it would be important to know if diverse GPCRs share similar structural organizations.

Most GPCRs are expressed at low levels in native tissue. Typically, investigators must overexpress them in cultured cells in order to study their structures and functions. The cannabinoid receptor 1 (CB_1_) is one of the highest-expressed GPCRs in the central nervous system, making it feasible to study in its natural state^1^.

CB_1_ is important for many biological functions such as pain, mood and memory^2–5^. Recent structural studies have revealed the isolated atomic arrangement of CB_1_ ^6,7^. However, these studies have primarily used X-ray crystallography or cryo-electron microscopy techniques that require high concentrations of well-purified proteins in detergent solution. CB_1_ structure has rarely been studied within cells in combination with signaling proteins, integral membrane proteins, or other membrane-associated proteins. Thus, the CB_1_ cellular structure remains incomplete, limiting our ability to fully exploit its function.

Super-resolution microscopy, an imaging method that overcomes the diffraction limit of conventional microscopy, has led to the discovery of the membrane-associated periodic skeleton (MPS) in the axons of neurons^8^. This highly ordered submembrane skeletal structure can play many roles in neuronal function, including acting as  a flexible mechanical support, organizing membrane protein distribution, and the development of axons and dendrites. The discovery of the MPS, and the many studies that have characterized cellular structures across different cell types^9,10^, demonstrate the power of super-resolution imaging for uncovering intracellular structures at the nano-scale. Recently, using a type of super-resolution imaging called STORM, Zhou et al.^11^ proposed a model where CB_1_ forms a periodic pattern when activated. They found a ~190 nm periodic pattern of CB_1_ in cultured hippocampal neurons exclusively under the administration of agonists. A previous study, using a similar imaging technology, also revealed distinct CB_1_ structures across different cell types in the brain, but showed no sign of a periodic pattern^12^. Thus, the intracellular organization of CB_1_ in neurons remains unclear.

Herein, by employing another type of super-resolution imaging, called stimulated emission depletion (STED), we systematically investigated the nano-structure of CB_1_ and other GPCRs in brain tissues and primary cultured neurons. Our results revealed a periodic structure of CB_1_ clusters along the axons of inhibitory interneurons. These CB_1_ clusters were organized into “hotspots” ~190 nm apart. Using dual-color STED imaging and cellular biology techniques, we demonstrated that the CB_1_ hotspots were associated with the MPS. The CB_1_ hotspots exhibited confined dynamics, which were reduced by receptor activation. Thus, our current studies demonstrate that the ~190 nm periodic structure of the cytoskeleton appears to  be the backbone for intracellular signaling to occur.

## Results

### CB_1_ exhibits semi-periodic hotspots in neurons

To study the structure of CB_1_ in vivo, we first demonstrated the specificity of our CB_1_ antibody labeling (Supplementary Fig. 1a, b). We found, in agreement with previous findings, that CB_1_ is mainly distributed in the axons of inhibitory interneurons, especially in those of cholecystokinin (CCK)-positive inhibitory interneurons (Supplementary Fig. 1c, d)^12^. CB_1_ did express much lower in myelinated axons and in the axons of excitatory neurons (Supplementary Fig. 1e, f).

**Fig. 1:**
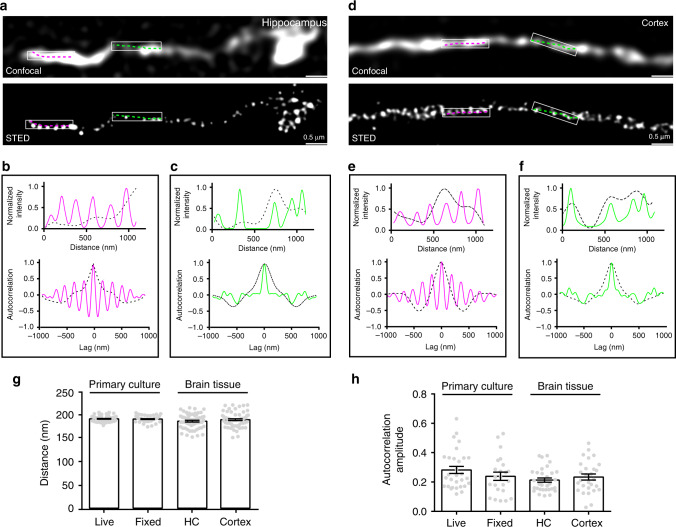
Periodic hotspots of CB_1_ of varying strength in axons from brain tissue. **a** Representative confocal and corresponding STED images of CB_1_ in the hippocampus of mature C57 mice. *N* = 3 biological replicates. **b**, **c** Intensity plotted along the lines in the box regions (top graph). Autocorrelation analysis for the confocal and STED images (bottom graph). **d**–**f** Similar to (**a**–**c**), but in the cortex region of mature C57 mice. *N* = 3 biological replicates. **g** Histogram of CB_1_ spacing in different samples, live and fixed hippocampal neurons, brain tissue of the hippocampus (HC) and cortex. Data are mean ± s.e.m. (*N* = 3 biological replicates; 70–120 axonal regions were examined per condition). *p* = 0.09, (no significance), one-way ANOVA. Actual spacing (from left to right), 192 ± 0.8 nm, 192 ± 0.8 nm, 187 ± 2 nm, 190 ± 2 nm. **h** Amplitude of the average autocorrelation functions calculated from randomly selected axon segments in different samples. *p* = 0.13 (no significance), one-way ANOVA. Actual autocorrelation amplitude (from left to right), 0.28 ± 0.02, 0.24 ± 0.03, 0.21 ± 0.01, 0.23 ± 0.02. Data in (**g**, **h**) are mean ± s.e.m. (*N* = 3 biological replicates; 70–120 axonal regions were examined per condition). Source data are provided as a Source Data file.

While we confirmed that CB_1_ was distributed in the axon shaft, the nature of its distribution was unknown. STED imaging is known to be well suited for studying the nano-scale structure of cellular components in fixed preparations^9,13^. Therefore, we undertook STED imaging, with a spatial resolution around 70 nm (Supplementary Fig. 2a), of immunolabeled hippocampal tissues to examine CB_1_ organizations along the axon shaft. CB_1_ displayed hotspots both with and without apparent periodicity within axons of the same axonal segment (Fig. 1a–c). We quantified the degree of periodicity using one-dimensional (1D) autocorrelation analysis by projecting the signals to the longitudinal axis of the axon and calculating the average 1D autocorrelation function over many axon segments^8,14^. The 1D autocorrelation amplitude, defined as the average amplitude of the peaks at ~190 nm, quantifies the degree of periodicity of the CB_1_ hotspots^8,14^.

To identify whether CB_1_ possess a similar distribution in other brain regions, we performed STED imaging in the cortex, and observed a similar semi-periodicity in neuron axons (Fig. 1d–f). The distance between these rhythmic hotspots was also ~190 nm in the cortex as it was hippocampus (Fig. 1g). Next, we imaged the structure of CB_1_ from cultured hippocampal neurons. We found that antibody-labeled CB_1_ exhibited hotspots with both high and low degree of periodicity in axons of cultured neurons (Supplementary Fig. 2b–d). These results are comparable to the in vivo results above (Fig. 1a–c).

To avoid artifacts caused by fixation procedures, we performed live super-resolution imaging on cultured neurons. To this end, we used structured illumination microscopy (SIM), a type of super-resolution imaging that is suitable for investigating the live structure of cellular molecules at high spatial resolution^15^. Using SIM microscopy, with a spatial resolution around 122 nm (Supplementary Fig. 2e), we revealed clusters of CB_1_ in cultured neurons, and those clusters appeared highly organized as hotspots in some regions of axons (Supplementary Fig. 2f–h). Again, CB_1_ exhibited both high and low periodic hotspots (Supplementary Fig. 2f–h). The spatial distance between periodic hotspots was ~190 nm, which was comparable to our results with antibody labeling in fixed preparations (Fig. 1g). Notably, the regularity of the CB_1_ structure in cultured neurons was higher than that in the brain (Fig. 1h). Therefore, we conclude that there is a commonality of the semi-periodic feature of CB_1_ in neurons, an unexpected characteristic of GPCRs in the cellular membrane.

### CB_1_ is associated with MPS in neurons

The semi-periodic organization of CB_1_ in axons raised two important questions, how is the CB_1_ semi-periodic organization formed and what are the complexes related to the semi-periodic organization of CB_1_? To answer the first question, we need to identify the components associated with the semi-periodic complex of CB_1_ in native states. To this end, we performed mass spectrometry (MS) experiments with modifications to increase the enrichment of membrane proteins in six different regions of the mouse brain (Supplementary Fig. 3). We found that CB_1_ was expressed at different levels in the six brain regions and that the expression level paralleled the AMPA specific glutamate receptor 1 and *N*-methyl-d-aspartate (NMDA) receptor (Supplementary Fig. 3a), which are known to crosstalk with CB_1_ in the central nervous system^16,17^. Furthermore, we observed the association of GPCR-related signaling molecules with CB_1_ (such as Gi, tyrosine-protein kinase and Fyn), indicating the reliability of our modified MS method (Supplementary Fig. 3b).

Next, we compared the expression extent of selected MPS components with CB_1_ expression. While different isoforms of ankyrin expressed across different functional domains of neurons, ankyrin-B (ankB) is predominantly distributed in the axons of neurons^14,18^. Our MS experiments indicated that the expression level of CB_1_ was highly correlated to that of ankB, but not ankyrin-R (ankR) (Supplementary Fig. 3a). Further, the expression level of CB_1_ was correlated to that of both αII-spectrin and βII-spectrin (Supplementary Fig. 3a) while other membrane proteins seem unrelated to the expression levels of CB_1_ (Supplementary Fig. 3c).

**Fig. 2:**
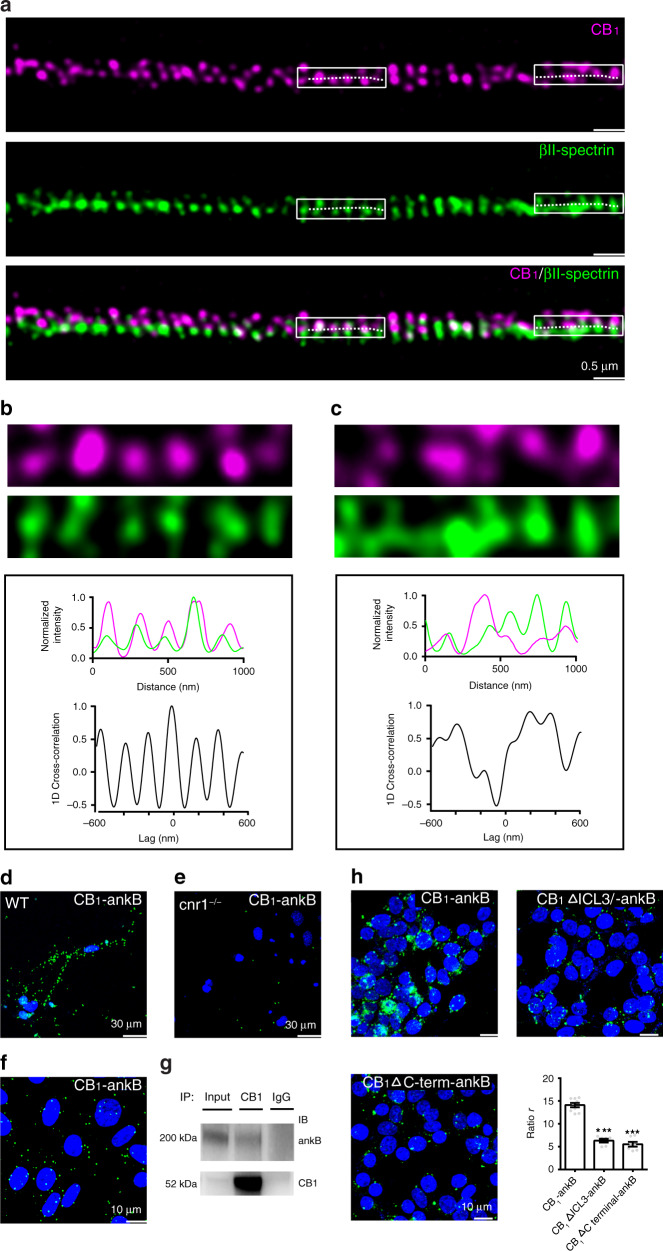
CB_1_ hotspots are connected with components of the MPS. **a** Two-color STED images of CB_1_ (magenta) and βII-spectrin (green) in the axons of cultured neurons. *N* = 3 biological replicates. **b**, **c** Top, enlarged images taken from white boxes from (**a**). 1D projection traces of βII-spectrin (green) and CB_1_ (magenta) signals along the axon are shown in the middle. 1D cross-correlation functions between the distributions of CB_1_ and βII-spectrin from CB_1_-positive axon segments are shown in the bottom. **d**–**f** PLA was performed in cultured neurons of WT mouse (**d**), *cnr1*^−/−^ mouse (**e**), and tetracycline-induced CB_1_ transfected CHO cells (**f**) with antibody of CB_1_ and ankB. Cell nuclei were stained with DAPI (blue). *N* = 3 biological replicates. **g** Immunoprecipitation of CB_1_ and ankB in CB_1_-CHO cells. Samples were processed for immunoprecipitation with either anti-CB_1_ or IgG control antibodies. Immunoprecipitates were immunoblotted with the anti-CB_1_ antibody (55kD) and anti-ankB antibody (220kD). *N* = 2 biological replicates. **h** PLA was performed in HEK-293T cells coexpressing ankB and different fragments of CB_1_, including wild type (CB1), CB_1_ with truncated ICL3 loop (CB_1_ΔICL3), or CB_1_ with truncated C-terminal (CB_1_ΔC-term). ratio *r* from left to right: 14.1 ± 0.5, 6.3 ± 0.4, 5.5 ± 0.4. Data are mean ± s.e.m. (*N* = 3 biological replicates; 25–50 imaging regions were examined per condition). ***p < 0.0001, statistical analysis was performed by unpaired two-tailed Student’s *t* test. Source data are provided as a Source Data file.

Earlier studies showed that MPS, like ankyrin, spectrin, displayed a highly coordinated pattern with an interval around 190 nm, which resembles the structure of CB_1_ in some regions of neurons revealed both by STED and SIM imaging experiments. Thus, consistent with our MS experimental results, there could be a tight association between CB_1_ and MPS components. To test the above view, we carried out two independent types of experiments, two-color STED imaging and proximity ligation assays (PLA)^19^. First, we visualized the spatial relation of CB_1_ and MPS molecules by dual-color STED imaging. The MPS was visualized through immunolabelling of the C-terminus of βII-spectrin, which is located at the center of each spectrin tetramer connecting adjacent actin rings^14,18^. We quantified the extent of colocalization between CB_1_ and βII-spectrin using 1D cross-correlation analysis to calculate the average 1D cross-correlation function between the two color channels over randomly chosen axon segments^8^. At periodic hotspots, CB_1_ displayed high colocalization with βII-spectrin while at nonperiodic clusters, CB_1_ displayed little colocalization with βII-spectrin (Fig. 2a–c). Previous studies demonstrated that ankB mediated the attachment of membrane proteins to the MPS, and is located in the middle region of each spectrin tetramer^18,20^. Thus, we reasoned that CB_1_’s interaction with ankB may organize the semi-periodic pattern of CB_1_ with the MPS. Indeed, at periodic hotspots, CB_1_ displayed high colocalization with ankB while at nonperiodic clusters, CB_1_ displayed little colocalization with ankB (Supplementary Fig. 4a–c). Furthermore, the spatial distance between periodic CB_1_ hotspots was comparable to both those of βII-spectrin and ankB (Supplementary Fig. 4d). These results unambiguously show that CB_1_ forms a semi-periodic complex associated with the MPS.

Then, to demonstrate the association between CB_1_ and the complex components identified, we carried out PLA experiments, another type of imaging assay that reliably detects the close physical distribution of two subjects^19^. It is known that α-adducin and βII-spectrin closely distribute in neurites. Indeed, in *cnr1*^−/−^ mouse, PLA signal with α-adducin and βII-spectrin was not changed (Supplementary Fig. 5a, b), indicating the reliability of PLA to detect proteins within a short distance. Then, we determined whether CB_1_ localizes closely with MPS components in cultured neurons. PLA signals of CB_1_ and ankB were observed in cultured primary neurons (Fig. 2d), but no PLA signals were detected in the *cnr1*^−/−^ mouse neurons (Fig. 2e). Upon the induction of CB_1_ in the presence of tetracycline, close physical distances were detected between CB_1_ and several MPS components, including ankB and βII-spectrin in CB_1_-CHO cell lines (Fig. 2f and Supplementary Fig. 5c, d). Together, these results from neurons and inducible CB_1_-CHO cell lines clearly illustrate the close physical distance between CB_1_ and MPS components.

In order to show that CB_1_ interacted with ankB in tetracycline-induced CB_1_-CHO cells, we performed immunoprecipitation experiments (Fig. 2g). To identify which parts of CB_1_ may mediate the interaction between CB_1_ and ankB, we truncated the third intracellular loop (ICL3) or C terminal of CB_1_. Notably, the PLA signals were significantly decreased in both conditions, suggesting that both regions, ICL3 and C-terminal, play a role in the interaction between CB_1_ and ankB (Fig. 2h), and that neither region alone is sufficient to mediate their interaction. Together, these results support the view that CB_1_ may form a complex with cytoskeleton-related proteins, such as ankB, αII-spectrin and βII-spectrin.

### CB_1_ structure in different compartments of axons

We examined whether the close relation between CB_1_ and cytoskeleton components locates in axons or dendrites of neurons. In order to achieve this, we first performed PLA labeling, and then immunolabeled either axons or dendrites with tuj1 or MAP2 antibody, respectively. Our results show that the PLA signal between CB_1_ and ankB substantially diminished after the second round of immunolabeling. We further explored the PLA signal between CB_1_ and spectrin, which can survive the immunolabeling procedure. Our results clearly showed that the PLA signal located in axons but not in dendrites (Supplementary Fig. 5e–f), indicating that CB_1_ is associated with MPS components specifically in axons.

Then, we used specific antibodies to label different regions of axons, such as axon initial segments (AISs) with βIV-spectrin antibody or presynaptic sites with VGAT antibody. Our results show that select AISs express CB_1_ with periodic pattern with an interval around 190 nm (Supplementary Fig. 5g, h, l–n) while some AISs did not express CB_1_ (Supplementary Fig. 5g, i). Interestingly, presynaptic sites with VGAT labeling did express CB_1_ (Supplementary Fig. 5j), but most were not periodic (Supplementary Fig. 5k). Thus, our results indicated that CB_1_ exhibited a specialized pattern in different regions of axons. Given that ankyrin and spectrin are uniformly distributed with a regular patter around 190 nm, there are likely some additional components mediating the CB_1_ distribution pattern in axons, such as the nonperiodic organization in presynaptic sites.

### CB_1_ displays confined dynamics in neurons

**Fig. 3:**
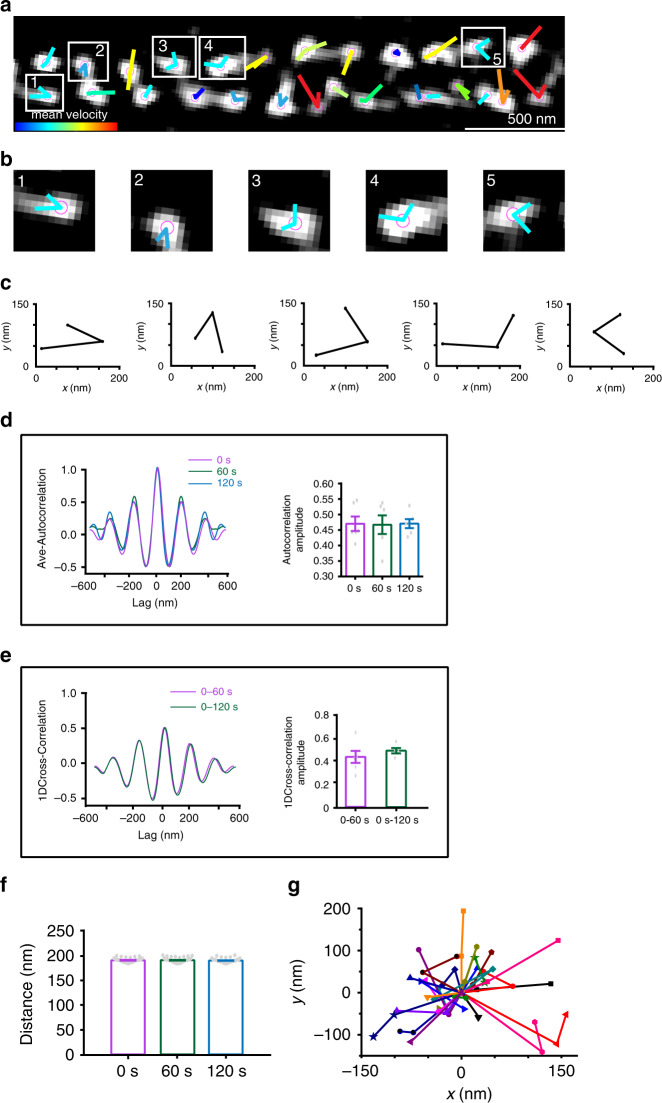
Periodic CB_1_ hotspots display stable dynamics revealed by live SIM imaging. **a** Representative live image of transfected CB_1_-RFP in the primary neuron (DIV 9–12) of the SD rat acquired by SIM. Individual CB_1_ hotspots are marked with purple balls, and locations of each individual time points are connected with lines. Line colors indicate trace indexes. *N* = 3 biological replicates. **b** Five CB_1_ hotspots shown in (**a**) with their relative locations. **c** Displacement changes from (**b**) are around 60–70 nm between neighboring time points. **d** Averaged autocorrelation analysis of CB_1_ distributions at different time points with the histogram of the autocorrelation amplitude. *p* = 0.99 (no significance), one-way ANOVA. Actual autocorrelation from left to right, 0.47 ± 0.02, 0.47 ± 0.03, 0.47 ± 0.02. **e** Averaged cross-correlation analysis between the neighboring frames (0 vs 60 s, 0 vs 120 s) showed similar distribution properties and histogram showing amplitude of average cross-correlation. *p* = 0.386 (no significance), statistical analysis was performed by unpaired two-tailed Student’s *t* test. Actual cross-correlation amplitude (from left to right), 0.45 ± 0.05; 0.51 ± 0.02. **f** The histogram of CB_1_ spacing across time points. *p* = 0.69 (no significance, one-way ANOVA). Actual spacing (from left to right), 192 ± 0.6, 192 ± 0.7, 191 ± 0.6 nm. Data in (**d**−**f**) are mean ± s.e.m. (*N* = 3 biological replicates; 70–120 axonal regions were examined per condition). **g** Traces of the individual CB_1_ hotspots over time. Source data are provided as a Source Data file.

CB_1_ connects to the MPS through ankB forming semi-periodic hotspots. As such, we would expect CB_1_ to display confined dynamics by interacting with the MPS through ankB. We investigated the dynamics of individual hotspots of CB_1_ with live SIM imaging. Neurons were ectopically expressed with a CB_1_-RFP fusion protein (RFP protein is fused to the end of CB_1_ C-terminal) and live SIM images were acquired 1 day after transfection. In order to identify whether CB_1_-RFP was recruited to the native site, we performed two-color STED imaging of CB_1_-RFP and βII-spectrin. CB_1_-RFP displayed highly periodic hotspots that colocalized with βII-spectrin, as well as nonperiodic clusters that did not colocalize with βII-spectrin (Supplementary Fig. 6a–c) These results were similar to the spatial distribution between CB_1_ and βII-spectrin (Fig. 2a–c), supporting that CB_1_-RFP is transported to CB_1_ native sites. We proceeded to examine the dynamics of CB_1_-RFP using live SIM imaging. Periodic CB_1_ hotspots were confined to movements around their starting positions displaying confined displacement changes (average 69 nm) between time frames (Fig. 3a–c). Averaged autocorrelation analysis of CB_1_ at different time frames showed a similar periodic distribution, indicating that CB_1_ clusters maintained their periodicity through time (Fig. 3d). The cross-correlation of neighboring time points was calculated showing that the average cross-correlation value peaked at zero point (Fig. 3e), indicating little or no systematic shift of the CB_1_ periodicity across different time frames. The periodic wavelength was around 190 nm at different time frames (Fig. 3f). We compiled the moving traces of individual hotspots of CB_1_ that clearly indicated confined movement both at short (2 min) (Fig. 3g) and longer (6 min) (Supplementary Fig. 7a–g) imaging time frames. The moving traces between the two time frames were comparable (Supplementary Fig. 7h), suggesting that CB_1_ displayed a confined movement around its anchoring point, likely mediated by ankB.

### Active CB_1_ associate with MPS and display less dynamics

**Fig. 4:**
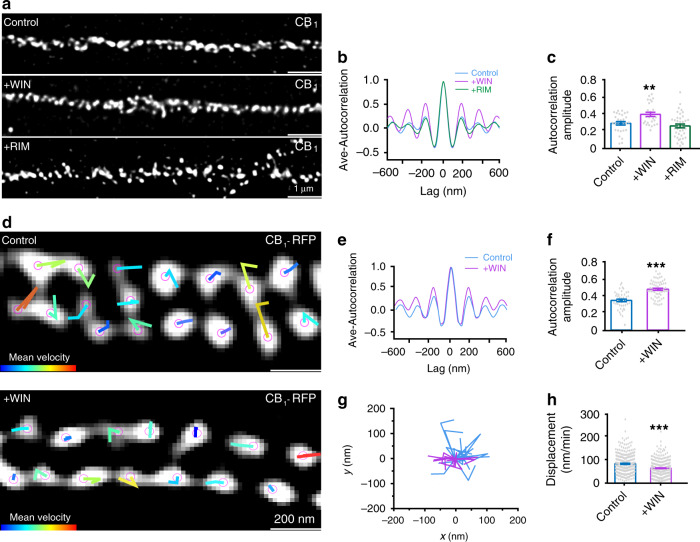
The dynamics of active CB_1_ hotspots. **a** Representative STED images of CB_1_ in primary hippocampal neurons for control, with WIN treatment (500 nM, 10 min) and RIM (1 µM, 10 min) treatment. *N* = 3 biological replicates. **b** Average autocorrelation analysis for the STED images of CB_1_ in different conditions. **c** The histogram of autocorrelation amplitude of CB_1_ (from left to right), 0.28 ± 0.02, 0.38 ± 0.02, 0.25 ± 0.02. *p* = 0.0003, **p < 0.01, one-way ANOVA. **d** Representative live image of transfected CB_1_-RFP in untreated neurons (top, “Control”), and neurons treated with WIN (bottom, “+WIN”) acquired by SIM. Individual CB_1_ hotspots are marked with purple balls, and locations of each individual time points are connected with lines. Line colors indicate trace indexes. *N* = 3 biological replicates. **e** Averaged autocorrelation analysis of CB_1_ distributions in live neurons. **f** The histogram of autocorrelation amplitude of CB_1_ without and with WIN treatment was shown. ***p < 0.0001, statistical analysis was performed by unpaired two-tailed Student’s *t* test. Actual autocorrelation amplitude (from left to right), 0.33 ± 0.02, 0.46 ± 0.01. **g** Traces the dynamics of CB_1_ hotspots without WIN treatment (magenta) and CB_1_ hotspots with WIN treatment (blue) over time. **h** The displacement of CB_1_ hotspots in different conditions. Control, 83.9 ± 2.5 nm/min. *N* = 291 spots. WIN, 64.6 ± 2.5 nm/min. *N* = 225 spots. ***p < 0.0001, statistical analysis was performed by unpaired two-tailed Student’s *t* test. Data in (**c**, **f**, **h**) are mean ± s.e.m. (*N* = 3 biological replicates; 70–120 axonal regions were examined per condition). Source data are provided as a Source Data file.

CB_1_ were displayed with coordinated clusters with an interval of around 190 nm upon WIN application with STED imaging the immunolabeled CB_1_ in the primary hippocampal neurons (Fig. 4a). Next, we imaged CB_1_ in the presence of antagonist to test whether blocking CB_1_ activity had an impact on its organization. Surprisingly, our results show that CB_1_ still exhibited a periodic pattern in some axons with CB_1_ antagonist, suggesting that the basal level activation of CB_1_ might not be necessary for the periodic organization (Fig. 4a–c). All raw STED images were included in Supplementary Fig. 8. To test the effect of CB_1_ activation on its dynamics, we imaged the dynamics of CB_1_ in the primary neurons transfected with CB_1_-RFP in the presence of CB_1_ agonist, WIN. Periodic CB_1_ hotspots were confined (Fig. 4d). CB_1_ displayed higher periodicity as reflected by the higher autocorrelation amplitude (Fig. 4d, e). Therefore, our results show that with WIN application, active CB_1_ moved around their original point to a lesser extent than nonactive CB_1_ (Fig. 4e).

Next, we used fluorescence recovery after photobleaching (FRAP) experiments to examine the recovery rate of CB_1_ after photobleaching to further study the dynamics of CB_1_ in culture neurons. We transfected the neurons with CB_1_-RFP. We then measured the half-time recovery rate of CB_1_. Our results showed that with WIN application the half-time recovery of CB_1_ was slower than in the control, while this effect was blocked with the application of CB_1_ antagonist (Supplementary Fig. 9a–c). The half-time recovery of CB_1_ was not affected by the application of agonists of other types of GPCRs, suggesting that the change was likely caused by CB_1_ activation (Supplementary Fig. 9c). Thus, our results show that CB_1_ displayed less dynamics with receptor activation.

### CB_1_ signaling is related to cytoskeleton

**Fig. 5:**
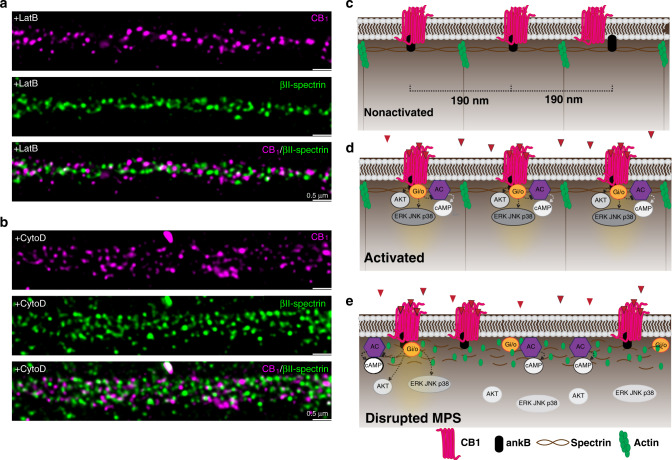
Schematic illustration showing CB_1_ forming dynamic peri-periodic hotspots to increase signaling efficiency. **a**, **b** Two-color STED images of CB_1_ (magenta) and βII-spectrin (green) in neurons treated with LatB (**a**) and CytoD (**b**). *N* = 3 biological replicates. **c** Without ligand binding, MPS sets the range for CB_1_ distribution. **d** Upon ligand binding, active CB_1_ are recruited to the MPS and become more periodic, making downstream AKT and ERK signaling more effective. **e** MPS degradation leads to less strong periodic clusters of CB_1_ and thus less downstream signaling.

To evaluate the dependence of CB_1_ periodic hotspots on the cytoskeleton, we treated cultures with either latrunculinB (latB) or cytochalasin D (cytoD) to disrupt cytoskeletal structure^8,14^. We found that following both latB and cytoD treatments, periodic hotspots of CB_1_ were no longer observed (Fig. 5a, b), suggesting that the cytoskeleton is important for maintaining the CB_1_ structure in neurons. Next, we tested whether the cytoskeleton affects CB_1_ intracellular downstream signaling. CB_1_ can activate both Akt and ERK1/2^21,22^. In primary neuron cells, pretreatment with latB resulted in decreased phosphorylation levels of both Akt and ERK1/2 in a dose-dependent manner (Supplementary Fig. 10a, b). Thus, these results suggest that the intracellular signaling of CB_1_ is dependent upon the MPS cytoskeleton (Fig. 5c–e).

## Discussion

Visualization of GPCRs in native tissue is necessary for understanding the intracellular organization of these receptors in real physiology. With the recent development of super-resolution imaging methods, we now can observe the organization of GPCRs at the cellular and tissue levels^12,11^. It is also important to visualize the live dynamics of GPCRs in order to understand potential functional changes in physiological and pathological conditions. Here, we used SIM to investigate the dynamics of CB_1_. Our findings with imaging probes likely reflect the dynamics of CB_1_ in neurons as both probes in live neurons displayed a spatial pattern similar to that of CB_1_ antibody in fixed neurons. Previous studies have shown that GPCRs form homodimers, heterodimers, or oligomers to affect their downstream signaling pathways^23–26^.

Uncovering the cellular structure of GPCRs has proven to be a challenging task, as several prior studies did not observe the semi-periodic organization of CB_1_ as we did^12,11^. An early study using STORM to characterize the CB_1_ structure in brain tissue did not observe any sign of organized CB_1_ pattern in axons. This could be due to the use of different antibodies, though most is likely because they did not focus on CB_1_ in the axonal shaft region, but instead focused on axonal boutons^12^. Intriguingly, Zhou et al.^11^ found that CB_1_ displays a nonperiodic structure without the application of WIN, an agonist of CB_1_, and becomes periodic upon the administration of WIN.

Our results using the same antibody used in Zhou’s experiments showed both periodic and nonperiodic hotspots in both culture neurons and native brain tissue. We observed the periodicity of CB_1_ clusters in live neurons to avoid artifacts caused by fixation procedures. Further, we found that active CB_1_ may behave differently than native ones, as CB_1_ displayed less dynamic and more confined movements upon WIN application. Our molecular biology and imaging results suggest that the ICL3 may participate in the association between CB_1_ and MPS components, such as ankB and spectrin. In the presence of WIN, CB_1_ can be activated and then some residues of ICL3 region are phosphorylated and contain more negative charges. By this way, more negative charged residues after receptor activation could attract and bind to the positive parts of the disordered domains of ankB and thus leading to a stronger connection between CB_1_ and ankB. This could explain the less movement of CB_1_ in the presence of WIN. The exact molecular mechanism underlying the distinct dynamics of CB_1_ is an important question and deserves further investigation.

Actually, Zhou’s data implied that there was weak CB_1_ periodicity and colocalization with spectrin at ~190 nm before agonist stimulation^11^. In the native state, we found that axons contained a semi-periodic pattern. Most synaptic sites did not exhibit periodic pattern, implying that the molecular mechanism to organize the synapses and CB_1_ complexes may be different. A small population of AISs had CB_1_ expression, while the others did not contain CB_1_ at all. This might be due to the fact that a small population of primary neurons in culture are inhibitory. The periodic distribution without application of WIN could be explained by the fact that constitutive released ligand, such as 2-AG, may activate CB_1_ to a certain extent and thus lead to the periodic pattern of CB_1_. However, in the presence of antagonist, CB_1_ still remained periodic in some regions of axons in primary culture neurons and its overall periodic pattern was not different. This suggests that the periodic organization of CB_1_ in native state might not be caused by constitutive released ligand in culture neurons, though we could not exclude this possibility in the brain tissue.

In conclusion, we found that CB_1_ is distributed in axons as organized hotspots separated by approximately 190 nm, in a similar spatial distribution to the MPS. Especially, CB_1_ tends to be more organized as periodic hotspots upon agonist application suggesting that active CB_1_ associate more strongly with the MPS. When a GPCR is activated by an agonist, it increases the kinetics of interaction with G proteins and conducts downstream signal transduction within hotspots, which are usually confined to the cytoskeletal and clathrin-forming grids^27^. Our results suggest that active CB_1_ is clustered into hotspots where G proteins or β-arrestin can easily collide with and form a relatively stable interaction with intracellular signaling components to increase signaling efficacy. Our results also indicate that CB_1_ are anchored to the MPS, likely by ankB, forming a fundamental structural unit that may be important for the proper function of neurons. These units likely form the structural basis for hotspots where signals transfer from extracellular to intracellular compartments. Our observation of periodic hotspots of CB_1_ along axon shafts, and the role of the cytoskeleton in CB_1_’s intracellular signaling, indicate a horizon for the study of structural−functional interaction in neurons.

## Methods

### Animals

C57BL/6 mice, Sprague−Dawley rat, CB_1_ knockout mice (Biocytogen) and Kunming (KM) mice (for knockout mice generation) were used in this study. Male C57BL/6 mice and CB_1_ knockout mice at 8–12 weeks were used. Animals were housed under a 12 h light/dark cycle at a room temperature of 22 ± 1 °C with 45% humidity, given ad libitum access to food and water. All experimental procedures were approved by the Institutional Animal Care and Use Committee of ShanghaiTech University, China.

### Generation of *Cnr1* knockout mouse

For *Cnr1* gene targeting, two sgRNAs were designed to target either the upstream or downstream region of its coding sequence by the CRISPR design tool (http://crispr.mit.edu) and screened for on-target activity using UCA^TM^ (Universal CRISPR Activity Assay, Biocytogen). PCR amplification was performed to add the T7 promoter sequence to the Cas9 mRNA and sgRNAs DNA template and then the T7-Cas/sgRNA PCR products were gel purified. They were used as the template for in vitro transcription with the MEGAshortscript T7 kit (Cat. No. AM1354, Life Technologies). The Cas9 mRNA and sgRNAs products were purified with MEGAclear kit (Cat. No. AM1908, Life Technologies) and eluted with RNase-free water.

C57BL/6 female mice and KM mouse strains were used as embryo donors and pseudo-pregnant foster mothers, respectively. Superovulated female C57BL/6 mice (3–4 weeks old) were mated to C57BL/6 stud males, and fertilized embryos were collected from the ampullae. Different concentrations of Cas9 mRNA and sgRNAs were mixed and co-injected into the cytoplasm of one-cell stage fertilized eggs. After injection, surviving zygotes were transferred into oviducts of KM albino pseudo-pregnant females and allowed to develop to term.

Mutant mice were genotyped to ensure the deletion of target CB_1_ segment. To mitigate off-targets effects, mutant mice were crossed into C57Bl/6 for five generations before being used for experimental purposes.

### Primary culture of rat neurons

Sprague−Dawley rats of either sex at P0 were used for culturing rat hippocampal or cortex neurons. Brain tissues were isolated and digested with papain (1 mg/mL; Sigma, P3125) at 37 °C for 30 min. The digested tissues were washed with Dulbeccoʼs Modified Eagle Medium (DMEM) solution (Hyclone, SH30243.FS) three times and then transferred to culture medium containing Neurobasal medium (Thermo Fisher Scientific, 21103049) supplemented with 2% (vol/vol) B27 supplement (Thermo Fisher Scientific, 17504044) and 1% (vol/vol) Glutamax (Thermo Fisher Scientific, 35050-061). The tissues were gently triturated until no chunks of tissue were left. Dissociated cells were then counted and plated onto poly-d-lysine-coated 12-mm coverslips (12-545-80, Fisher Brand) or 29 mm Glass (#1.5 cover glass) bottom dishes (D29-20-1.5-N, Cellvis). Neuronal cultures were maintained in the culture medium in a humidified atmosphere with 5% CO_2_ at 37 °C. One-half of the medium was changed every 3 days to maintain neuron viability.

### Fluorescence labeling of neurons

Cultured neurons were fixed with 4% (w/v) paraformaldehyde (PFA) in phosphate-buffered saline (PBS) for 15 min at 14 day in vitro (DIV 14). After a complete wash with PBS, the samples were then permeabilized and blocked in the blocking buffer (10% v/v donkey serum, 0.2% v/v Triton X-100 in PBS) for 1 h at room temperature, and subsequently stained with one or two primary antibodies in the incubation buffer (1% donkey serum, 0.1% Triton X-100 in PBS) overnight at 4 °C. The samples were washed in PBS and then stained with secondary antibodies as described above in the incubation solution for 1 h at room temperature. After incubation, samples were washed with PBS. Neuron samples were mounted with ProLong Gold (P36930, Life Technology) and imaged.

### Perfusion and immunostaining of brain sections

Briefly, adult male C57BL/6 mice and CB_1_ knockout mice were anesthetized with sodium pentobarbital (40 mg/kg, i.p.) with no avoidance response to foot pinch. They were then perfused with normal saline (at 37 °C) and subsequently by ice-cold 4% PFA for fixation. Brains were post-fixed in 4% PFA for 4 h, then dehydrated in 30% sucrose. Brains were frozen at −80 °C and then sectioned at 20-μm-thick with the freezing microtome (Leica CM1950) for immunofluorescence labeling.

For the fluorescence immunostainings procedure, brain sections were rinsed in PBS, permeabilized, and then blocked with blocking solution (3% w/v donkey serum and 0.3% v/v Triton X-100 in PBS) for 2 h at room temperature and then overnight at 4 °C with the primary antibody in 0.1% Triton X-100 and 1% serum in PBS. After washing with PBS, sections were incubated with the secondary antibody in 0.1% Triton X-100 and 1% serum in PBS for 2 h at room temperature. After incubation, sections were washed with PBS. Sections were mounted with ProLong Gold mounting medium for following imaging.

### STED imaging

Confocal and STED images were obtained at a Leica TCS SP8‐3X gated STED system (Leica Microsystems) equipped with a pulsed white light laser (WLL, tunable from 470 to 670 nm) for excitation, a 592 nm CW laser (MPB Communications), a 660 nm CW laser (MPB Communications), and a 775 nm pulsed laser (Onefive) for depletion. The system includes a ×100 objective lens (Leica, HC PL APO CS2 ×100/1.40 oil), two HyD detectors, and the TCS SP8 time-gated system. Each time before imaging, the depletion laser was co-aligned with the excitation laser. By STED effect, the lateral fluorescence was filtered out, leaving the remaining fluorescence in the center to be detected.

For single or dual color 2D imaging on the primary neuron culture, samples were excited by the WLL at 488 nm (Alexa Fluor 488-labeled βII-spectrin or ankB) and 561 nm (Alexa Fluor 555-labeled CB1) and depleted by 592 and 660 nm laser, respectively. The emission spectrums were set at 493–550 nm and 566–600 nm accordingly. Channels were recorded sequentially. Acquisition parameters were optimized as follows: logical size, 1024 ×1024 pixels; pixel size, 23 nm; scan speed, 400 Hz; scan direction, unidirectional; line average 4, no frame average; optical zoom factor 5, pinhole 1 airy disc; detector gain, 100%; HyD time gating, 0.3–6 ns for confocal and 1–6 ns for STED; excitation power 10–30 pW; STED power, 11–17 mW for 592 nm and 5–8 mW for 660 nm.

For single-color imaging on fixed brain slices, CB1 was labeled with Alexa Fluor 647, excited by the WLL at 633 nm and depleted by 775 nm laser. The emission spectrum was set at 657–700 nm. In order to reduce the adverse effect of aberration and scatter caused by the thickness of tissue, only those processes in close proximity (<1 μm in *z* axis) and parallel to cover glasses were chosen to acquire 2D images. The HyD time gating was set at 0.3–6 ns for confocal and 0.5–6 ns for STED, and the power of 633 nm laser and 775 nm laser was 20–30 pW and 7–8 mW, respectively. Other acquiring parameters were similar to the above.

Deconvolution of STED images was performed by Huygens software (Scientific Volume Imaging) with the Huygens classical maximum likelihood estimation (CMLE) deconvolution algorithm and theoretical Point Spread Functions. The deconvolution was performed by using Deconvolution Wizard. Deconvolution parameters were set to match the refractive index (RI) of the mounting media (RI of ProLong Gold polymerized until complete dry was 1.47) and the RI of the objective oil (1.518). Detailed deconvolution parameters were as follows, background, automatic estimation; estimate mode, lowest; area radius, 0.7; deconvolution algorithm, CMLE; maximum iteration, 40; signal-to-noise ratio, 7–10; quality threshold, 0.001; iteration mode, optimized; PSFs per brick, one PSF; brick layout, auto.

To measure the spatial resolution of the STED imaging microscope, 20 nm fluorescent microspheres (F8887, Thermo Fisher Scientific) were used to measure the performance of microscope. Beads were diluted 100-fold with ddH2O and then sonicated in order to disperse bead aggregates. 1 μL beads were fully spread on a 0.17-um-thick and PLL-coated coverslip. After about ten minutes with those beads dried, coverslips were mounted on the slide with a thin layer of mounting media (ProLong Gold). The slide was kept at room temperature until the ProLong Gold was fully dried. The microspheres were excited by a 561 nm laser and depleted by a 660 nm laser. The acquiring parameters were optimized as follows: logical size, 1024 ×1024 pixels; pixel size, 11 nm; scan speed, 400 Hz; scan direction, unidirectional; line average 4, no frame average; optical zoom factor 10; pinhole 1 airy disc; detector gain, 100%; HyD time gating, 0.3–6 ns for confocal and 1–6 ns for STED; excitation power, 30–40 pW; STED power, 15 mW. Intensity profiles across the center of beads were plotted in the FIJI software, and the FWHM (Full width at half maximum) was calculated by fitting with a Gaussian function^28^.

### Live SIM imaging the structure of CB_1_ in neurons

SIM images were obtained with the GE DeltaVision OMX microscope, equipped with a 568-nm laser (Coherent), a ×60 objective lens (Olympus, PL Apo N ×60/1.42 oil), and the scientific CMOS camera (Acquisition pixel size, 82 nm at ×60 objective; PCO edge). 3D-SIM mode was acquired with a fixed 512 × 512 pixel size and an optical section space of 0.125 μm. Fifteen images were taken with three illumination angles and five phase shifts for each *Z*-section. Images were taken in fast 286 MHz mode.

Primary neurons were transiently transfected using lipofectamine 2000 (Invitrogen) at DIV 9–12 (2 µg plasmid DNA/20 mm dish). Briefly, neurons were mixed with transfection complexes containing CB_1_-RFP plasmid DNA and lipofectamine 2000 at a ratio of 1:2 in MEM medium (Thermo Fisher Scientific, 11090081) for 2 h, then subsequently incubated with the culture medium. One day after transfection, neurons expressed CB_1_-RFP were imaged under the live 3D-SIM mode. The cells were incubated with Neurobasal medium (without phenol red, Thermo Fisher Scientific, 12348017) at 37 °C and supplied with 5% CO_2_ mixed with 95% air during the imaging process. To obtain images with minimized spherical aberration and optimized illumination contrast, immersion oil with different refractive index (RI) were systematically optimized for each individual sample until a symmetrical point spread function was achieved. Usually the oil with RI 1.520 was chosen. To reduced phototoxicity, the illumination laser intensity and the exposure time were carefully adjusted to a minimum value (10% of maximum laser intensity; exposure time, 6–10 ms) and only 1 μm in depth (eight optical sections) were acquired. The time series of 3D-SIM images were acquired six frames with 1 or 3 min acquisition interval. An ultimate focus system was used to maintain the sample *Z* position, regardless of the mechanical and thermal changes during the acquisition.

The raw SIM images were reconstructed by the OMX SI reconstruction tool available in softWoRx (GE). Channel-specific OTF files, channel-specific K0 angles, Wiener filter constant (0.001), and bias offset (65) were used during the reconstruction. Widefield images were generated by averaging phase steps from SIM raw images.

To detect the SIM microscope resolution, the bead samples were made similarly to those for STED, but use Vectashield (H-1000, Vectorlabs) as mounting medium instead of Prolong Gold. Slides were sealed with nail polish and imaged. The imaging procedure was similar to the live SIM imaging. FWHM was determined with the same method to STED.

After SIM reconstruction, the images were processed in the FIJI software. The rigid image drift during acquisition was corrected with “Correct 3D drift” plugin^29^. Images were maximum-intensity projected and the linearly contrast was adjusted using Fiji software (National Institutes of Health). For the analysis and display, only those examples that could be tracked and did not bleach more than 20% for consecutive three time frames were selected. Fiji TrackMate was then used to analyze the dynamics of CB_1_ hotspots in these images^30^. In TrackMate, the difference of Gaussian (DoG) detector was set with an estimated spot diameter of 80 nm and an appropriate fluorescence intensity as the threshold to detect all the individual CB_1_ hotspots. The Simple Linear Assignment Problem (LAP) tracker with a linking maximum distance of 200 nm, a gap-closing maximum distance of 200 nm, and a gap-closing maximum frame gap of 2 was used for tracking cells through the time course images.

### In situ PLAs

The protocol for PLA can be found on Duolink in situ PLA detection Kit (Sigma) following the instructions of the supplier with slight modifications^31^. Neurons at DIV 14–18 were fixed and incubated with primary antibodies overnight as described in the section of immunofluorescence staining. On the next day neurons were washed 3× with Phosphate-Buffered Saline, 0.1% Tween® 20 Detergent (PBST). All remaining steps were carried out in a 37 °C humidified chamber. To detect CB_1_−cytoskeleton protein interaction, a mixture of equal amounts of anti-CB_1_ antibody directly linked to a plus PLA probe and cytoskeleton protein antibody (like ankyrin B or βII-spectrin) directly linked to a minus PLA probe was used. Neurons were incubated with a pair of PLA probes diluted 1:10/each in 1× blocking solution for 1 h, before washing 3× at 10 min each with 1× Washing Buffer-A. Ligase was diluted 1:40 into 1× Ligation buffer and added to neurons for 0.5 h ligation, followed by 3 × 5 min washes with 1× Washing Buffer-A. Polymerase was then applied 1:80 to neurons in 1× amplification stock for 2.5 h. Neurons were washed 2 × 10 min with 1× Washing Buffer-B, 1 × 10 min with 0.01× Washing Buffer-B, and 1 × 10 min with PBST. Neurons were fixed again by 4% PFA for 10 min to better retain the PLA signals before incubated with axonal primary antibody like MAP2 or Tuj1 at room temperature for 2 h. Then neurons incubated with fluorescent secondary antibody for 1 h at room temperature before mounting with Mounting Medium with 4′,6-Diamidino-2-Phenylindole, Dihydrochloride (DAPI). The samples were observed in a Nikon confocal microscope equipped with an apochromatic ×60 oil-immersion objective (N.A. 1.4), and a 405, a 488 and a 561 nm laser line. For each field of view, a stack of three channels (one per staining) and 10−15 *Z* stacks with a step size of 1 μm were acquired. Images were opened and processed with Fiji software. The ratio *r* (number of red spots/number of cells containing spots) was determined considering a total of 300−600 cells from 6 to 10 different fields.

### Fluorescence recovery after photobleaching

FRAP experiments on the culture neuron transfected with CB_1_-RFP were also performed on the GE DeltaVision OMX microscope with a ×60, 1.47 NA objective. Culture neurons were transfected with CB_1_-RFP 1 day before imaging. Prior to the experiment, the medium was half changed to Neurobasal without phenol red. The cells were maintained at room temperature with 5% CO_2_ mixed with 95% air supplied during the whole imaging process. Each FRAP image was taken with a fixed laser intensity and exposure time (7–10 ms per frame) and 1-s interval. No images were acquired during the bleach period. Putative axonal regions transfected with CB_1_-RFP were chosen and an image stack of 1–2 μm with 0.4-μm interval in in *Z* axis were acquired. Ultimate focus system was also used to maintain the stability of the microscope. Six pre-bleach images were taken followed by bleaching a 1-μm-diameter circle region for 0.1 ms with a strong laser intensity. Immediately after bleach, post-bleach images were taken until the recovery reached a complete steady state. Images were maximum projected and processed in Fiji. Briefly, FRAP data were quantified using Fiji software. During image processing, the background level was subtracted from each frame to quantify the recovery of fluorescence. Average fluorescence intensity of the bleached area was corrected for additional bleaching during regular imaging and was normalized to pre-bleach intensity. The image fluorescence over time was normalized to initial fluorescence (average fluorescence value from the 5-s imaging period immediately before photobleaching, defined as 100%) and immediately after bleaching (0%). Analysis of the recovery fitting curves and the half-time recovery were carried out with the Fiji macro (http://imagej.net/Analyze_FRAP_movies_with_a_Jython_script)^32^.

### Western blot

Primary neurons were either treated or not treated with the indicated ligands for the times noted, rinsed with ice-cold PBS, and lysed by the addition of 100 μL of ice-cold RIPA lysis buffer (Beyotime) with protease inhibitor cocktail (Roche). Cellular debris was removed by centrifugation at 13,000 × *g* for 15 min at 4 °C, and the amount of protein was quantified by BCA protein assay kit (Pierce). Protein samples were separated on 10% SDS-PAGE gels (Bio-Rad) and transferred onto PVDF (polyvinylidene fluoride) membranes (Millipore, Billerica, MA, USA). The membranes were blocked with 5% non-fat dry milk for 2 h at RT and incubated overnight at 4 °C with rabbit anti-phospho-ERK1/2 (1:2000, Cell Signaling), anti-phospho-Ser473-Akt (1:2000, Cell Signaling), anti-ERK1/2 (1:2000, Cell Signaling), and anti-AKT (1:2000, Cell Signaling) antibodies. The membranes were then incubated with HRP-conjugated secondary antibody (1:1000; Pierce) for 2 h at room temperature. Signals were visualized using enhanced chemiluminescence (ECL, Pierce, Rockford, IL), and captured by the ChemiDoc XRS system (Bio-Rad Laboratories, CA). Phosphorylated ERK1/2 or phosphorylated Akt levels were normalized for differences in loading using protein band intensities for total ERK1/2 or AKT.

### Immunoprecipitation

Following tetracycline induction, CB_1_-CHO cells were washed with ice-cold PBS and suspended in immunoprecipitation (IP) buffer containing (in mM), 50 Tris-HCl, 120 NaCl, 0.5% Nonidet P-40 and protease cocktail (pH = 7.5). The lysate was sonicated, centrifuged at 13,000 × *g* for 20 min at 4 °C, and the resulting supernatant was incubated with the rabbit anti-CB_1_ (CST, 93815) antibody for 20 min at 4 °C. Immuno-complex was incubated with Protein A-Magnetic beads overnight on a rotating wheel at 4 °C from the precleared supernatant with anti-CB_1_ antibody covalently coupled to Protein G-Magnetic beads. The pellet was then washed 5 times in wash buffer containing (in mM), 20 Tris-HCl, 100 NaCl, 1 Ethylenediaminetetraacetic acid (EDTA), 0.5% Nonidet P-40 (pH = 8.0). The beads were to wash away any proteins nonspecifically bound to the beads. The immunoprecipitates were mixed with the loading buffer and resolved by SDS-PAGE. Western blots were performed with relevant antibodies. Rabbit IgG was used as a negative control.

### Mass spectra in brain tissues

#### Mouse brain tissue preparation

Preparation of mouse brain membrane fractions was performed according to a previous study^33^. Briefly, six brain regions (olfactory bulb, cerebral cortex, cerebellum, hippocampus, midbrain, and spinal cord) were obtained from 9-week-old C57BL/6 wild-type male mice. Brain regions from three mice were pooled and homogenized in the buffer of 300 mM sucrose, 0.5% bovine serum albumin (BSA), 100 mM EDTA, 30 mM Tris/HCl, pH 7.4 with protease inhibitor (Roche). Crude membrane fractions were isolated from the homogenate by ultra-centrifugation at 160,000 × g at 4 °C for 1 h. The membrane pellet was solubilized in 4% SDS and 100 mM dithiothreitol (DTT) in 100 mM Tris/HCl, pH 7.6, denatured and reduced at 95 °C for 3 min. Protein concentration was determined using the BCA assay. For each brain region, protein sample preparation was conducted in duplicate.

#### Protein digestion

The SDS-assisted digestion of membrane proteins was performed according to methods described previously^34^. Briefly, 50 μg of protein was diluted in 8 M urea, 50 mM NH_4_HCO_3_, and exchanged to the same buffer using the 30 KDa MWCO centrifugal filter unit (Satorious, Germany) by centrifugation at 13,000×*g* for 20 min. The following centrifugation steps were performed under the same conditions. Subsequently, 100 μL of 50 mM iodoacetamide in 8 M urea, 50 mM NH_4_HCO_3_ was added and incubated at room temperature in darkness for 30 min, then followed by centrifugation. The concentrate was diluted with 200 μL 50 mM NH_4_HCO_3_ and centrifuged again, this step was repeated twice. Proteins were digested with trypsin (Promega, Madison, USA) at an enzyme-to-protein ratio of 1:100 (w/w) at 37 °C for 3 h, followed by the addition of trypsin at 1:50 (w/w) and incubation at 37 °C overnight. After acidification, the protein digest was desalted with C18-SepPak columns (Waters, Milford, USA) and lyophilized under vacuum.

#### NanoLC-MS/MS analysis

The nanoLC-MS/MS analysis was conducted on an EASY-nLC 1000 connected to Orbitrap Fusion mass spectrometer (Thermo Fisher Scientific, USA) with a nano-electrospray ionization source. The eluted peptides were separated on an analytical column (200 mm × 75 μm) in-house packed with C18-AQ 3 μm C18 resin (Dr. Maisch, GmbH, Germany) over a 130-min gradient at flow rate of 300 nL/min. For a pooled sample from all six brain region protein digests, a data-dependent (DDA) acquisition method was first employed with the following parameters, resolution of 60,000 used for survey scans; the mass range pf 300−1700 m/z; an AGC target value of 4E5; and maximum ion injection time of 50 ms. Up to 12 dynamically chosen and most abundant precursor ions were fragmented. The MS/MS scans were acquired at an Orbitrap resolution of 30,000 (AGC target value 1E5, maximum ion injection time 50 ms).

In order to achieve the accurate quantification of selected proteins in the membrane fractions, we developed parallel-reaction-monitoring (PRM) MS assays for all proteins of our interest based on the protein identification results from the DDA experiment^35^. The PRM acquisition method started with a full scan event followed by targeted MS/MS for specific peptides from the proteins of interest. Major parameters for the MS/MS event in Orbitrap were a resolution of 30,000; an AGC target value of 2E5; and maximum injection time of 100 ms. Peptide precursor ions in different brain region protein digests were monitored in the PRM assay by scheduling an inclusion list of each precursor with an isolation window of 1.6 m/z and retention time shift of 2 min.

### MS data processing

Mass spectra from the DDA experiment were processed using Proteome Discoverer 2.1 against the Uniprot mouse sequence database. The search parameter included cysteine carbamidomethylation as a fixed modification and oxidation of methionine as variable modification. Precursor ion mass tolerance was set to 10 ppm and fragment ion tolerance was 0.02 Da. Trypsin was set as the specific enzyme and two missed cleavages were allowed. The required false discovery rate (FDR) was set to 1% at the peptide and protein level.

For PRM data analysis, the Skyline software (v3.7.0) was used for targeted peptide quantification with settings specified by the software instruction^36^. Only *b*- or *y*-product ions with *m/z* values greater than the precursor were selected to quantify the peptides. All the transitions were validated using the mProphet algorithm in Skyline advanced peak picking model that restricts the FDR to <1%. The peptide quantification was derived from the sum of the peak areas of 3−6 product ions for selected peptides. Protein intensity was based on the summed MS responses of one to three unique peptides of the corresponding protein.

### Quantification and statistical analysis

All image data were first processed with Fiji software (National Institutes of Health). Images were resized with the Bicubic interpolation and the brightness and contrast were linearly adjusted. To quantitatively analyze the distribution properties of CB_1_, segmented lines across the structures were drawn, the intensity profiles along the lines were measured and further analyzed in Matlab (MathWorks, Inc.). Individual fluorescence peaks were found and the distance between the neighboring peaks were calculated and pooled together. For distribution pattern analysis, autocorrelation and cross-correlation were performed on the fluorescence intensity profiles. The correlation curve was pooled and averaged from many randomly selected lines. All intensity, distance and correlation data were plotted using Graphpad prism (Graphpad Software, Inc.), and all figure layouts were composed in Illustrator (Adobe Systems, Inc.).

Results were reported as mean ± s.e.m. Statistical analysis of the data was performed using a Student’s *t* test, one-way ANOVA. Statistical significance was set at *p* < 0.05.

## Supplementary information

Supplementary Information

## Data Availability

All data are available upon reasonable request. Source data are provided with this paper.

## Source data

Source Data
