# Supplementary material for: Organized cannabinoid receptor distribution in neurons revealed by super-resolution fluorescence imaging
Source: Nat Commun. 2020 Nov 11;11:5699. doi: 10.1038/s41467-020-19510-5 (PMC7659323; doi:10.1038/s41467-020-19510-5)
Supplement: Supplementary file 1 — Supplementary Information [file 41467_2020_19510_MOESM1_ESM.pdf]

Organized cannabinoid receptor distribution in neurons revealed by super-resolution fluorescence imaging

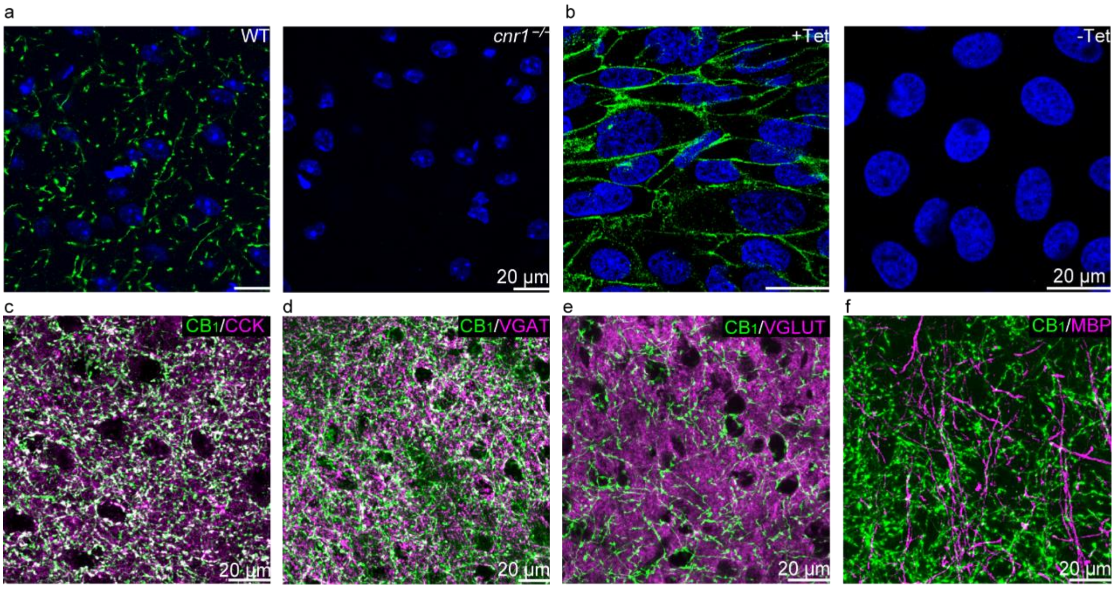

Supplementary Figure 1 Confocal imaging reveals the antibody specificity of CB_1
a Immunostaining of CB_1 (green channel) in wild-type mouse (left panel) and *cnr1*$^{-/-}$ mouse (right panel). **b** Immunostaining of CB_1 with the antibody used in a CB_1-CHO cell line with (left) and without (right) tetracycline application. **c-f** Representative fluorescent images of, (**c**) CCK; (**d**) VGAT; (**e**) VGLUT; and (**f**) MBP, all colocalized with CB_1. N = 3 biological replicates each.

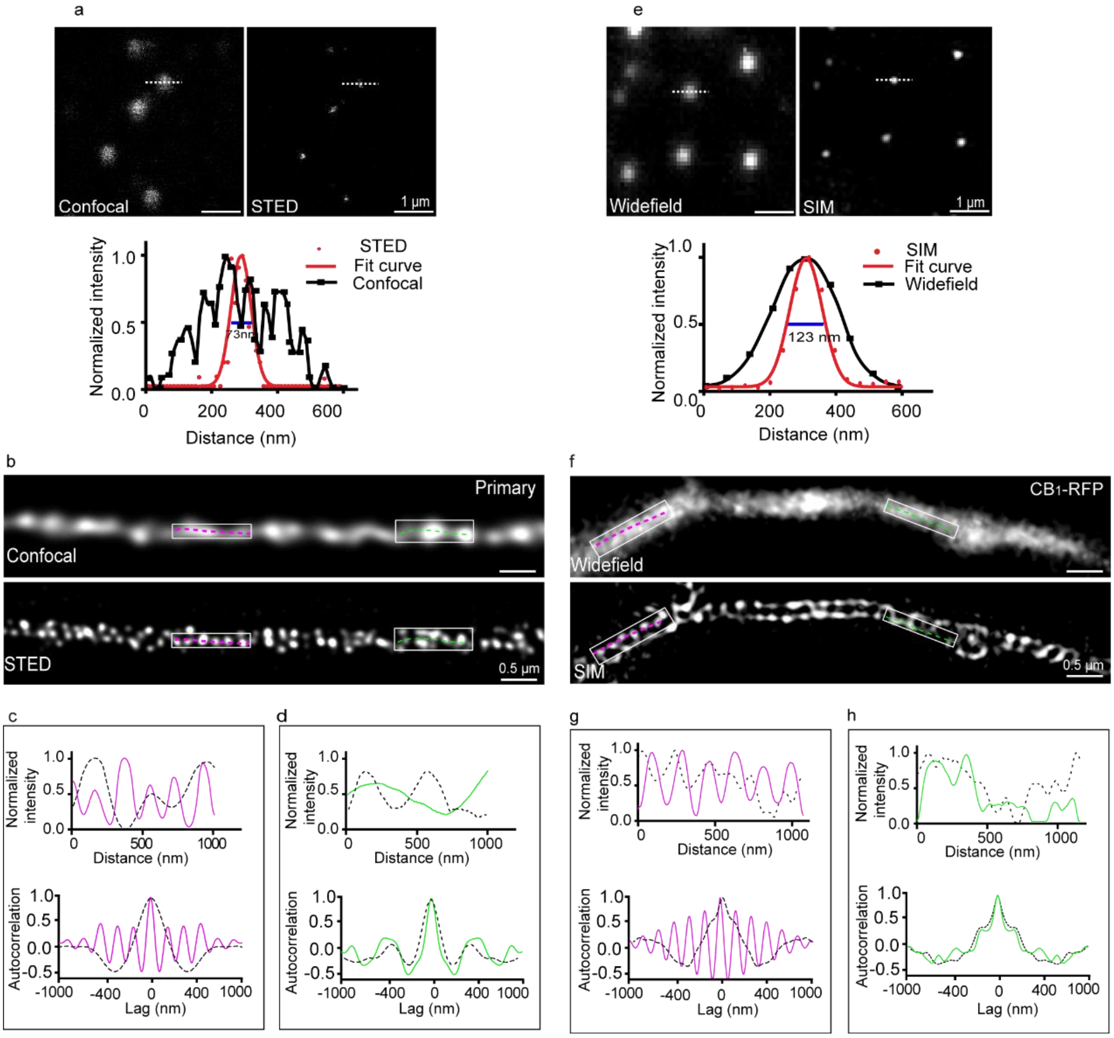

Supplementary Figure 2 The semi-periodic hotspots of CB_1 in cultured neurons. **a** Confocal and STED imaging of 20nm beads with the 568 nm excitation laser. Intensity profiles of one bead (dashed line) were plotted, and the FWHM was determined by fitting with a Gaussian function (70.2±1.6nm, N=53 fluorescent beads). **b** Representative confocal and corresponding STED image of CB_1 labeled by antibody in culture hippocampal neurons. N = 3 biological replicates. **c-d** Corresponding intensity and autocorrelation analysis along the lines in the box regions in **b**. **e** Reconstructed widefield and SIM imaging of 20nm beads with the 568 nm excitation laser. Intensity profiles of one bead were plotted, and the full width at half maximum (FWHM) was determined by fitting with a Gaussian function (122.2±1.2 nm, N=51 fluorescent beads). **f** Representative wild field and corresponding SIM images of CB_1 labelled CB_1-RFP in culture hippocampal neurons. N = 3 biological replicates. **g-h** Corresponding intensity and autocorrelation analysis along the lines in the box regions in **f**. Source data are provided as a Source Data file.

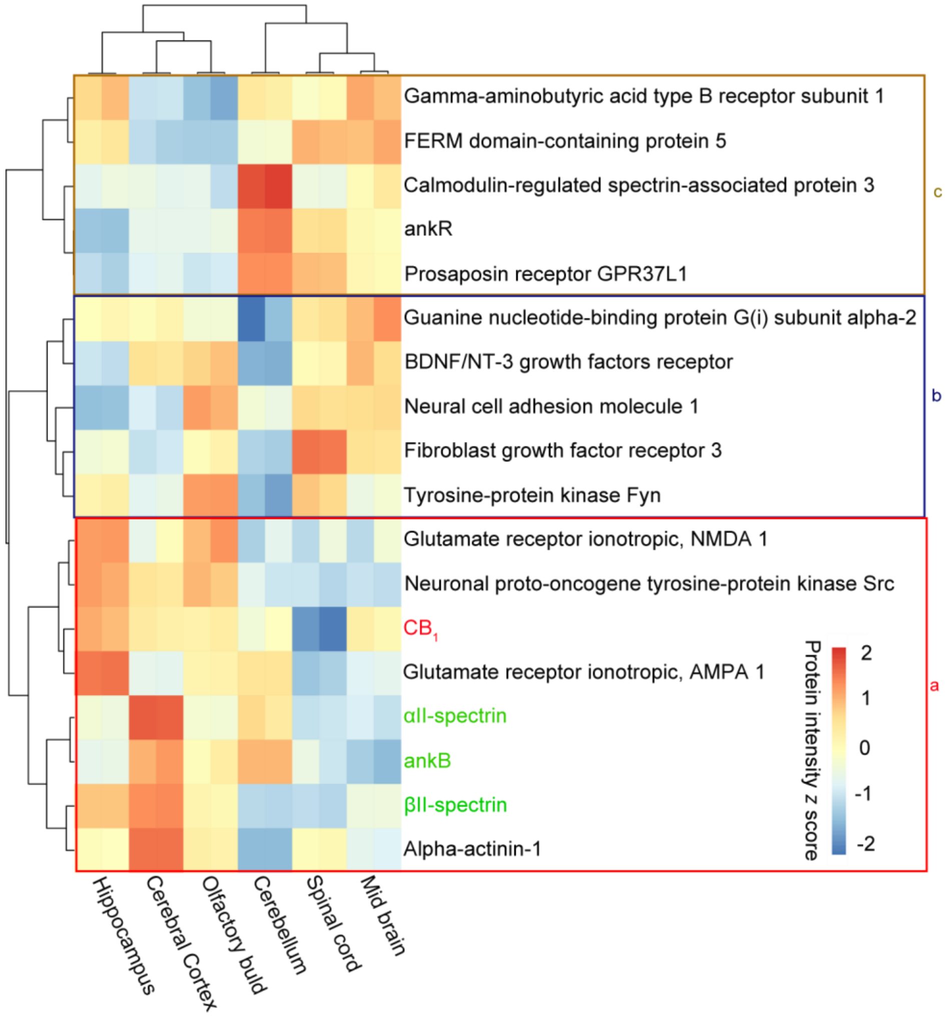

Supplementary Figure 3 CB_1 and associated proteins revealed by MS. Heat map of three groups of selected protein expressed across different brain regions (n = 2 for each region). Heat map of z-scored protein abundances of the differentially expressed proteins (determined with PRM MS assays) after unsupervised hierarchical clustering revealed the correlation on the expression level of CB_1 to cytoskeleton-related proteins **(a)**, receptors, signaling molecules **(b)**, and other less likely related proteins **(c).**

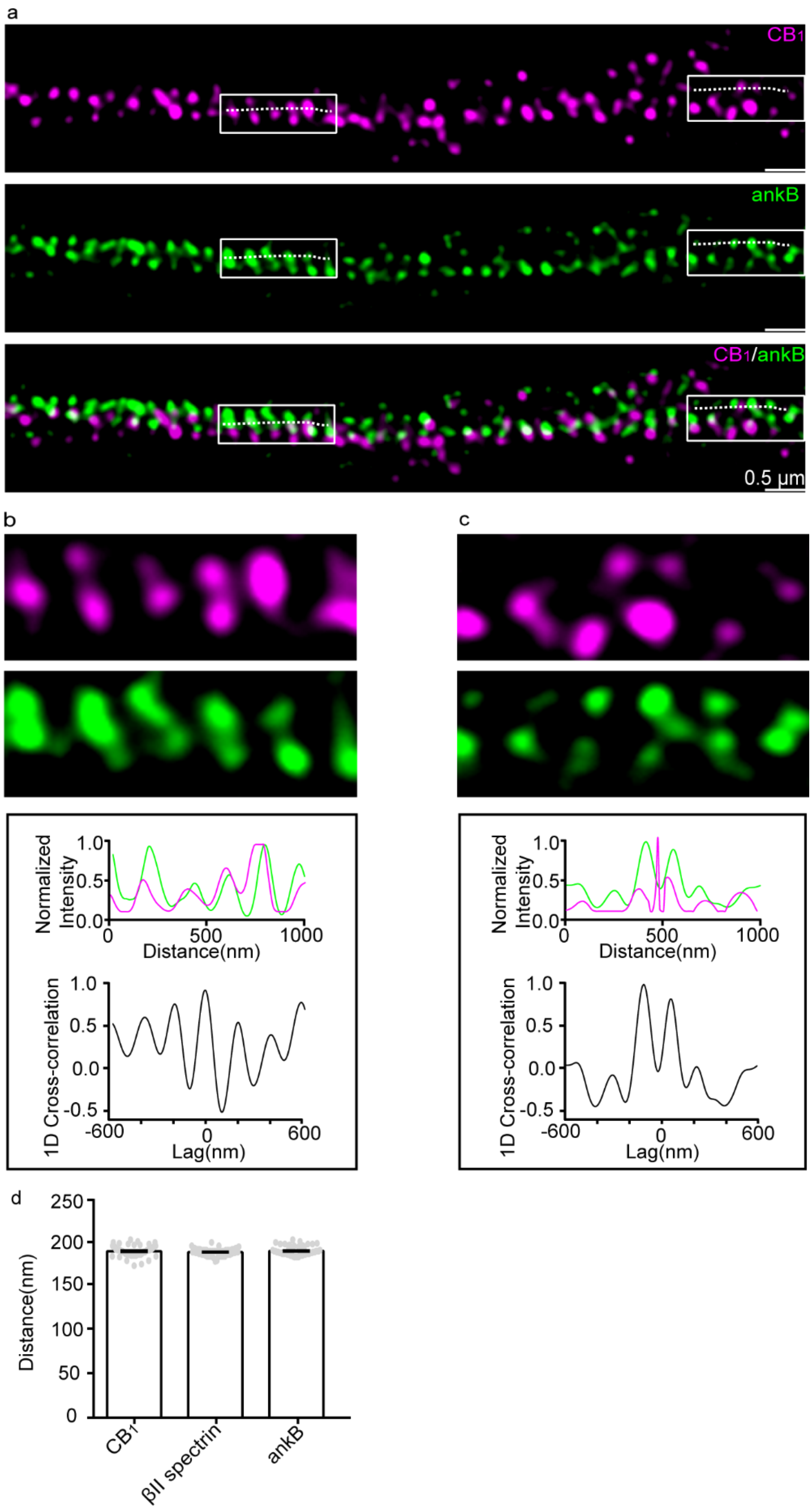

Supplementary Figure 4 Spatial relationship between CB_1 and ankB revealed by STED. **a** Two-color STED image of CB_1 (magenta) and ankB (green) in the axons of

culture neurons. N = 3 biological replicates. The white boxes represent regions enlarged in **b** (left box) and **c** (right box). **b-c** Enlarged regions from **a** (top). Normalized intensity and 1D cross-correlation analyses between the distributions of CB_1 and ankB from white lines in **a** (bottom). **d** Comparison of the periodic spacing between CB_1 and cytoskeleton components in the primary neurons. Data are mean ± s.e.m. (N = 3 biological replicates; 70-120 axonal regions were examined per condition). P = 0.32, no significance ($p > 0.05$), one-way ANOVA. Actual spacing (from left to right), 192 ± 0.8 nm, 191 ± 0.5 nm, 192 ± 0.7 nm. Source data are provided as a Source Data file.

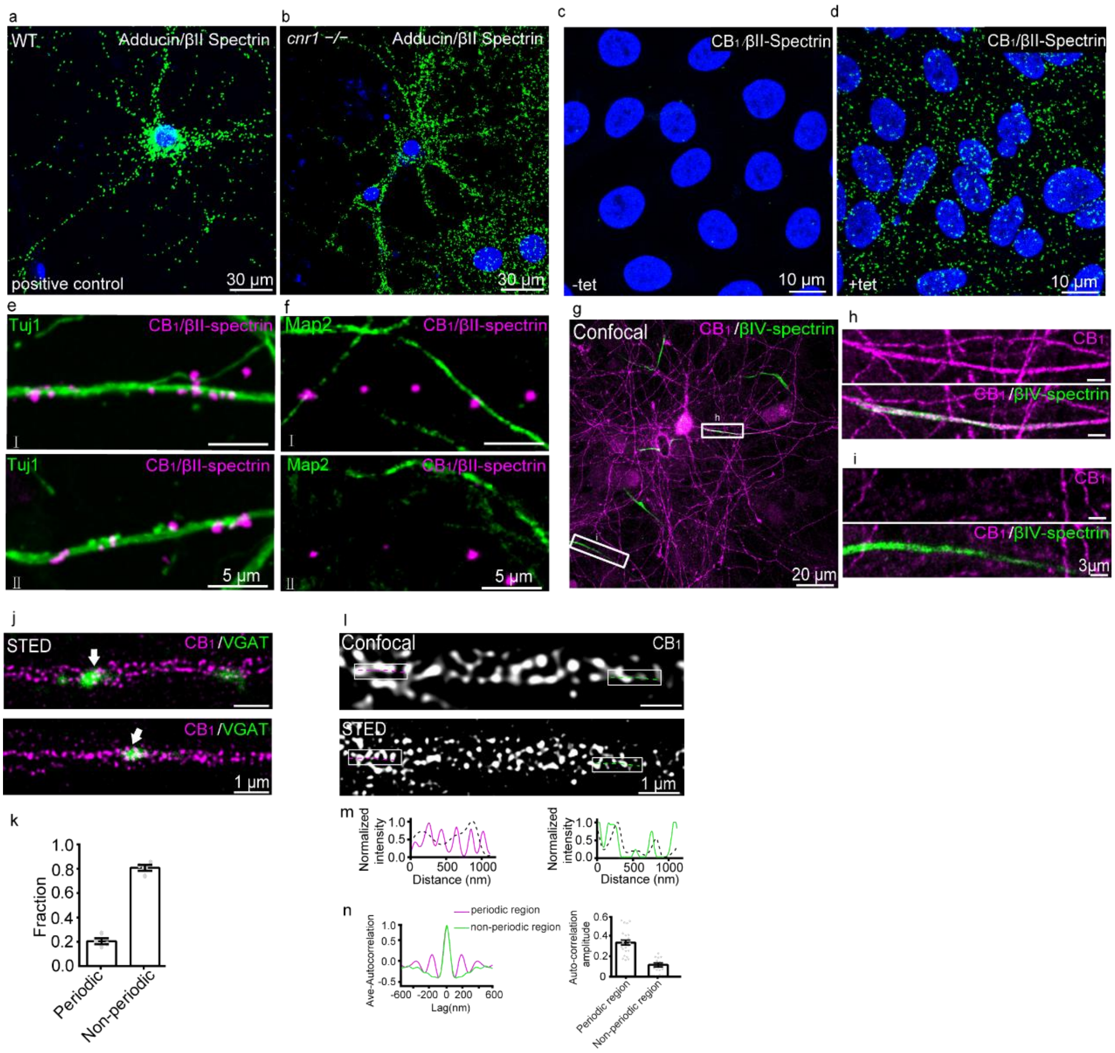

Supplementary Figure 5 PLA signals were distributed in axons. **a-b** PLA signals were detected in the cultured neurons of both WT (**a**) and *cnr1*$^{-/-}$ mouse (**b**) with antibody of CB_1 and adducin, which have certain interactions. N = 3 biological replicates. **c-d** PLA analyses of CB_1 and βII-spectrin in CB_1-CHO cells in the absence (**c**) and presence (**d**) of tetracycline application. N = 3 biological replicates. **e** PLA signals (magenta) were detected in Tuj1(green) positive neurites of cultured neurons (DIV12), (I)-(II) represent different regions in the axons of culture neurons. **f** PLA signals (magenta) were detected in Map2 (green) negative neurites of cultured neurons (DIV12), (I)-(II) represent different regions in cultured neurons. N = 3 biological replicates for **e-f**; 30-50 axonal region were examined per condition). **g** Confocal fluorescence image of hippocampal primary neuron culture labeled for CB1 (magenta) and AIS marker βIV-spectrin (green).. **h** Magnification of the boxed region in g that CB1-positive expression at AIS region. **i** Magnification of the boxed region in g that CB1-negative expression at AIS region. N = 3 biological replicates for g-i. j Representative STED images of CB_1 in axons that contain presynaptic boutons marked by VGAT (magenta). N = 4 biological replicates. **k** the percentage of periodic and non-periodic organization of CB_1 in axonal synaptic sites (N = 4 biological replicates; 50 to 100 axonal bouton regions

were examined per condition). l Representative confocal and STED images of CB1 in the AIS. Fraction from left to right, 0.2±0.02, 0.8±0.02. Data are mean ± s.e.m. N = 3 biological replicates. m Intensity plotted along the lines in the box regions in l. n Averaged autocorrelation analysis of CB1 distributions at AIS region with the histogram of the autocorrelation amplitude. Actual auto-correlation from left to right, 0.33±0.02, 0.11±0.02. Data are mean ± s.e.m. N = 3 biological replicates; 24 periodic region and 13 non-periodic region at AIS were examined. Source data are provided as a Source Data file.

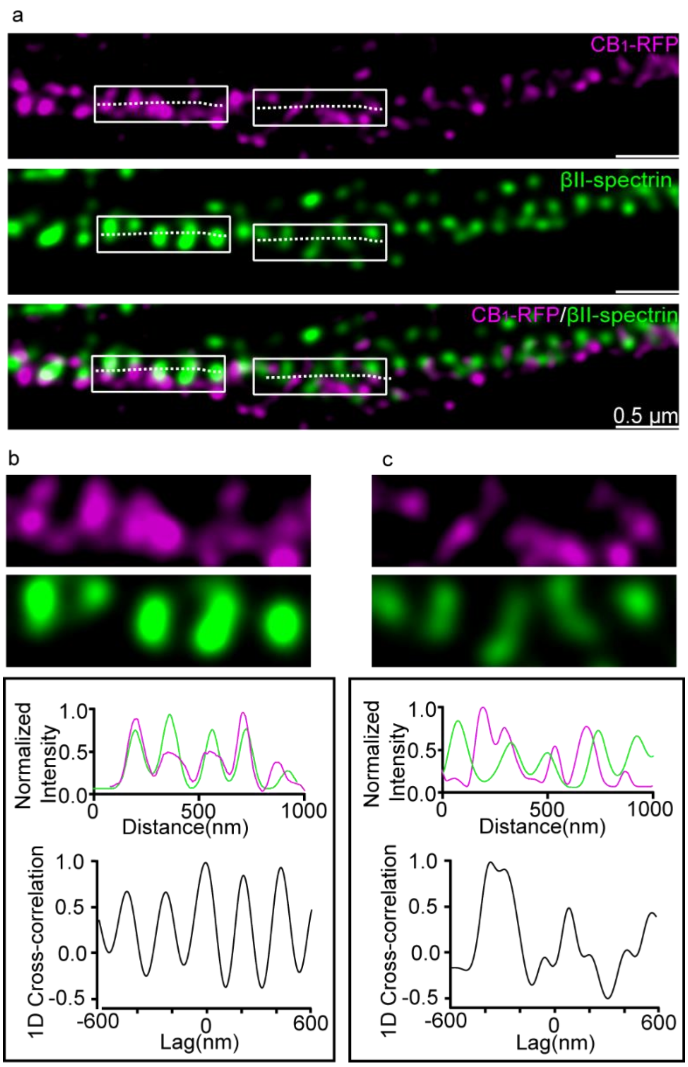

Supplementary Figure 6 Spatial relationship between CB_1-RFP and βII-spectrin revealed by STED. **a** Two-color STED image of CB_1-CFP (magenta) and βII-spectrin (green) in the axons of cultured neurons. N = 3 biological replicates. The white boxes represent regions enlarged in **b** (left box) and **c** (right box). **b-c** Enlarged regions from **a** (top). Normalized intensity and 1D cross-correlation analyses between the distributions of CB_1-CFP and βII-spectrin from white lines in **a** (bottom).

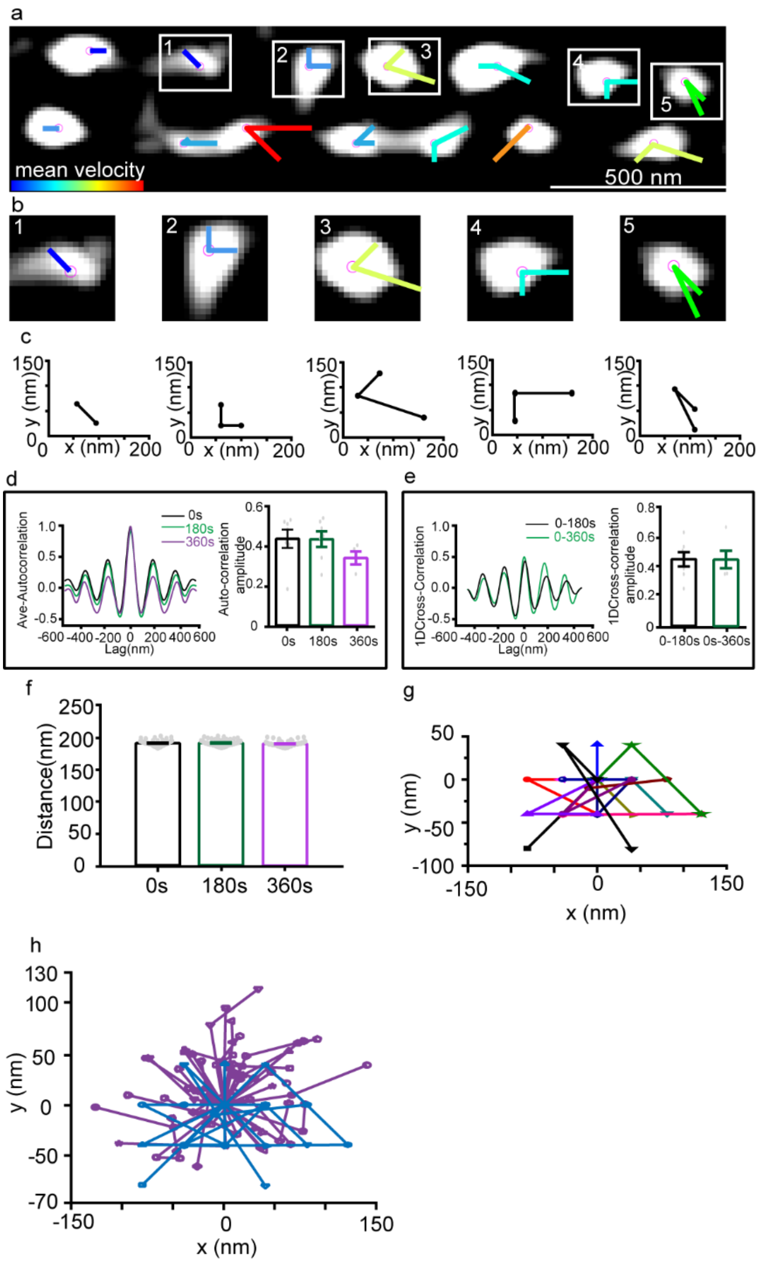

Supplementary Figure 7 The dynamics of CB_1 hotspots revealed by live SIM imaging. **a** Representative live image of transfected CB_1-RFP in the primary neuron (DIV 9-12) of the SD rat acquired by SIM taken at an interval of 3 mins. Each CB_1 hotspot was marked with a purple dot, and its location at each individual time point is connected by a line. The color of line indicates the trace index. N = 3 biological replicates. **b-c** The five CB_1 hotspots shown in **a** and their relative locations. Displacements are around 60-70 nm between neighboring time points. **d** Averaged autocorrelation analysis of CB_1 distributions at different time points with the autocorrelation amplitude shown on the right. There was no statistical difference between time points. P = 0.31, no significance ($p>0.5$), one-way ANOVA. Actual autocorrelation amplitude (from left to right), 0.43 ± 0.04, 0.43 ± 0.04, 0.34 ± 0.03. **e** Averaged cross-correlation analysis between

neighboring frames (0s-180s, 0s-360s) showed similar distribution properties (left) and amplitude (right). P = 0.99, no significance ($p>0.5$), statistical analysis was performed by unpaired two-tailed Student's t-test. Actual cross-correlation amplitude (from left to right), 0.45 ± 0.05, 0.45 ± 0.06. **f** CB_1 spacing across time points. P = 0.71, no significance, one-way ANOVA. Actual spacing (from left to right), 192 ± 0.6 nm, 192 ± 0.7 nm, 192 ± 0.6 nm. **g** Dynamics of the individual CB_1 hotspots over time. **h** Overlay of the dynamics of the individual CB_1 hotspots over time taken at interval of 1 min (purple) or 3 min (green). The moving distance did not increase over time during imaging, suggesting that individual hotspots displayed a confined dynamic around its original point. Data in **d**, **e** and **f** are mean ± s.e.m (N = 3 biological replicates; 70-120 axonal regions were examined per condition). Source data are provided as a Source Data file.

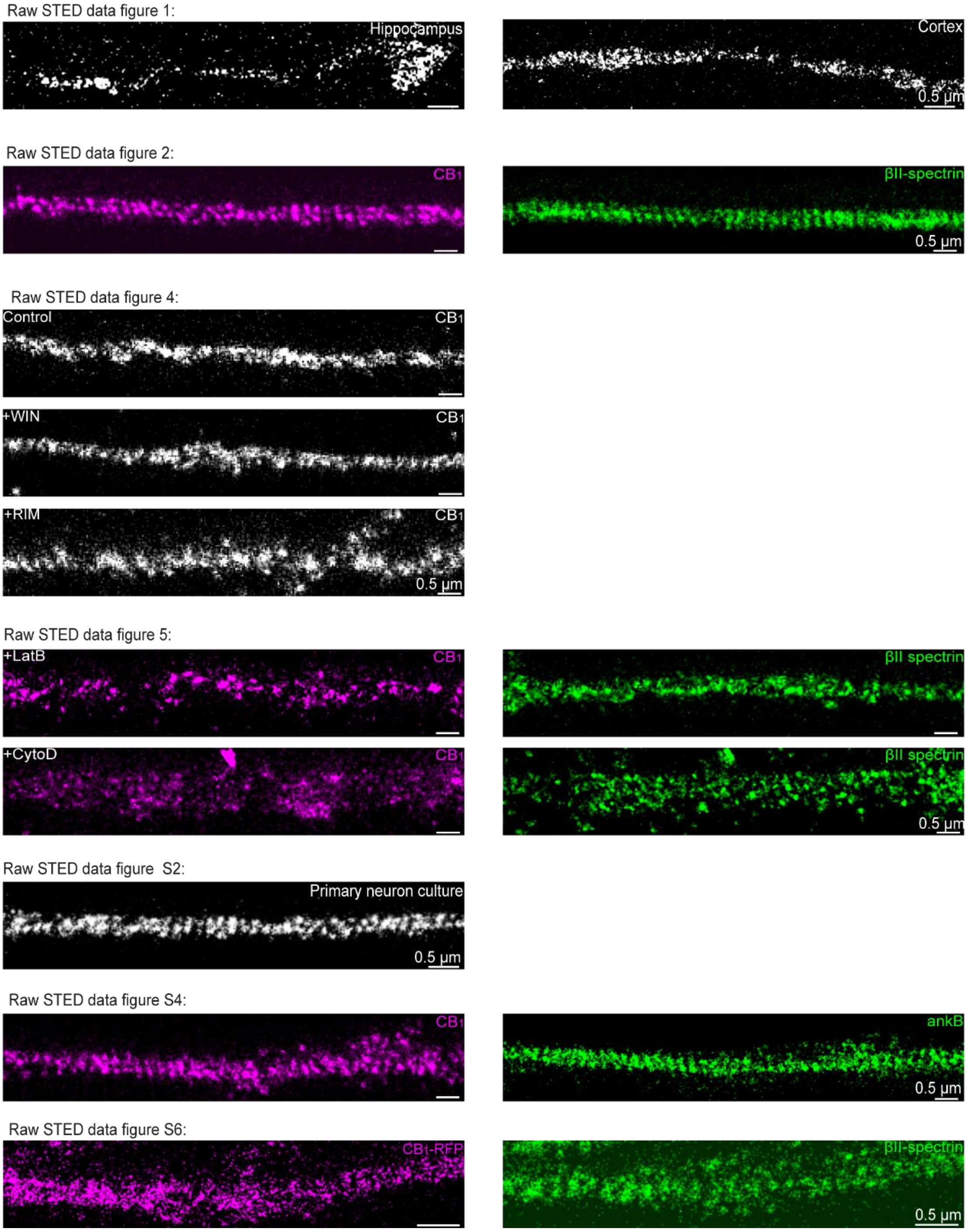

Supplementary Figure 8 Raw STED images from figures are shown here. As only parts of the data could be shown in the paper, representative images are chosen to illustrate the typical structure.

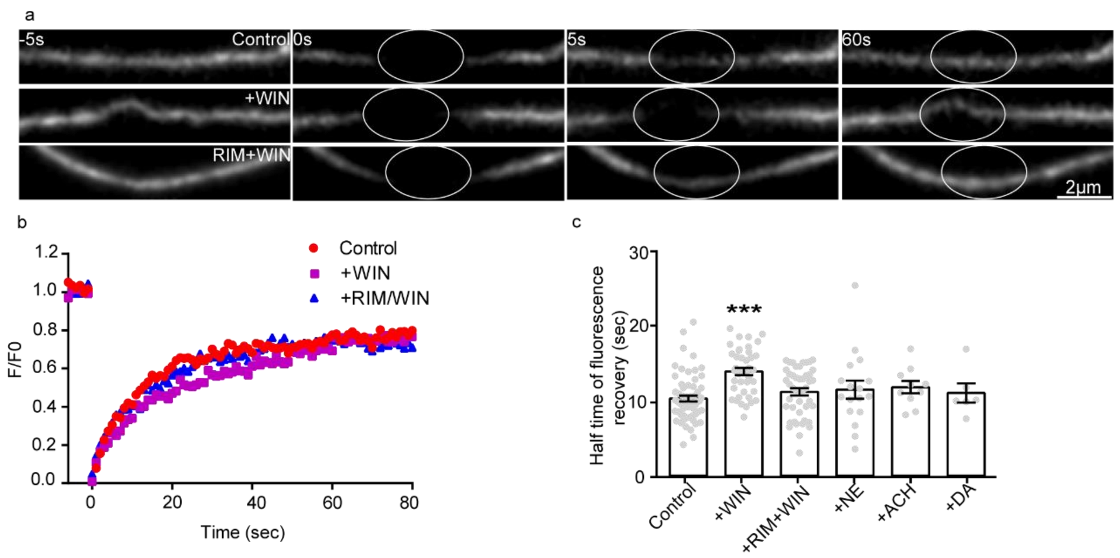

Supplementary Figure 9 Activated CB1 displays lower mobility a Images from representative CB1-RFP FRAP experimental time course in basal (Ctrl), WIN 55,212–2 (WIN, 500 nM) or Rimonabant (RIM, 1 μM)+WIN 55,212–2 (WIN, 500 nM)-treated conditions. N = 3 biological replicates. **b** The time course of CB1-RFP fluorescence after photobleaching in control, WIN or Ri+WIN conditions. Values represent means from at least 10 cells for each. **c** Bar graph of the means (± s.e.m.) of half-time of recovery for CB1-RFP (N=61 for Control; Actual half time of fluorescence recovery from left to rifht, 10.44 ± 0.4, 14.10 ± 0.5, 11.34 ± 0.5, 11.64 ± 1.2, 11.97 ± 0.8, 11.19 ± 1.3; N=40 for WIN; N=46 for RIM+WIN; N=16 for NE, 1 μM; N=10 for ACH, 1 μM; N=6 for DA, 1 μM; N represents axonal segment. ***p<0.0001 for Ctrl vs WIN, unpaired two-tailed Student's t-test). Source data are provided as a Source Data file.

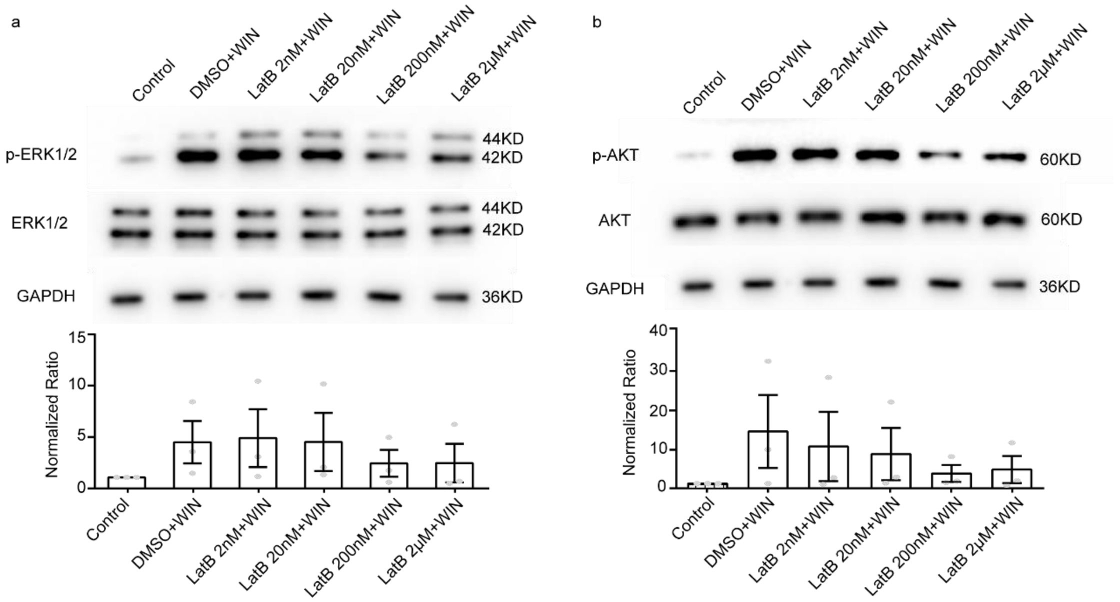

Supplementary Figure 10 CB_1 signaling is related to the cytoskeleton

a Western blot analysis for ERK1/2 and phosphorylated ERK1/2 in whole-cell lysates from primary neuron cells treated with different concentrations of latB. N = 3 biological replicates. Normalization from left to right, 1, 4.23±1.9, 4.62±2.7, 4.26±2.7, 2.27±1.2, 2.30±1.8. Data are mean ± s.e.m. **b** Western blot analysis for Akt and phosphorylated Akt in whole-cell lysates from primary neuron cells treated with different concentrations of latB. N = 3 biological replicates. Normalization from left to right, 1, 14.045±9.1, 10.28±8.67, 8.39±6.5, 3.52±2.1, 4.5±3.4. Data are mean ± s.e.m. Source data are provided as a Source Data file.
